# Human Monoclonal Antibody-Based Therapy in the Treatment of Invasive Candidiasis

**DOI:** 10.1155/2013/403121

**Published:** 2013-06-26

**Authors:** Francesca Bugli, Margherita Cacaci, Cecilia Martini, Riccardo Torelli, Brunella Posteraro, Maurizio Sanguinetti, Francesco Paroni Sterbini

**Affiliations:** Institute of Microbiology, Università Cattolica del Sacro Cuore, Rome, Italy

## Abstract

Invasive candidiasis (IC) represents the leading fungal infection of humans causing life-threatening disease in immunosuppressed and neutropenic individuals including also the intensive care unit patients. Despite progress in recent years in drugs development for the treatment of IC, morbidity and mortality rates still remain very high. Historically, cell-mediated immunity and innate immunity are considered to be the most important lines of defense against candidiasis. Nevertheless recent evidence demonstrates that antibodies with defined specificities could act with different degrees showing protection against systemic and mucosal candidiasis. Mycograb is a human recombinant monoclonal antibody against heat shock protein 90 (Hsp90) that was revealed to have synergy when combined with fluconazole, caspofungin, and amphotericin B against a broad spectrum of *Candida* species. Furthermore, recent studies have established an important role for Hsp90 in mediating *Candida* resistance to echinocandins, giving to this antibody molecule even more attractive biological properties. In response to the failure of marketing authorization by the CHMP (Committee for Medicinal Products for Human Use) a new formulation of Mycograb, named Mycograb C28Y variant, with an amino acid substitution was developed in recent years. First data on Mycograb C28Y variant indicate that this monoclonal antibody lacked efficacy in a murine candidiasis model.

## 1. Introduction

Largest scientific effort to develop antibody-(Ab-) based therapies has focused on diseases where the humoral immune system was known to contribute in a crucial way to host defense.

Infectious diseases caused by viruses or by encapsulated bacteria such as pneumococcus and meningococcus have represented the major targets for antibody therapy [1–3].

Despite broad-spectrum of antibiotic therapy has almost completely replaced serum therapy for bacterial diseases, to now, hyperimmune human immunoglobulins are used to treat many viral diseases including those caused by cytomegalovirus respiratory syncytial virus, hepatitis A virus, hepatitis B virus, and others [4, 5], highlighting the concept that antibodies-based therapy remains an effective means of treatment.

The humanized monoclonal Ab (mAb) palivizumab, targeted the RSV F protein, is effective for the prevention of severe respiratory disease in high-risk infants and immunocompromised adults and represents the only one licensed mAb for an infectious disease [6].

The enormous potential offered by monoclonal antibodies as therapeutic agents has been only slightly exploited by the field of infectious diseases, contrary to what happened to areas of medicine like oncology and that of autoimmune diseases where the use of monoclonal antibodies has provided an outstanding contribution to current therapies [7, 8].

Another area where antibodies therapy has definitely brought the leading therapeutic choice is the neutralization of animal venoms [9].

On the other hand, recent works have determined that mAbs could be effective even against microbes, such as fungi or intracellular pathogens, for which the principle studies do not clearly defined a role played by humoral immunity [10].

Macrophage, NK cells, and neutrophils related to cell-mediated immunity and nonspecific cellular immunity are generally believed to be the main protagonists for the primary defenses against fungi [11]. The importance of cellular defense mechanisms for protection against fungi is supported by the clinical observation that most invasive fungal infections occur in individuals with defective cellular immunity. As a matter of fact, in the field of medical mycology it is generally accepted that cellular immunity is essential for successful host defense against fungi [12].

How long antibodies are actually involved in the defense against fungal infections remains a controversial issue [13]. The literature shows a rather heterogeneous orientation regarding the actual importance of humoral immunity for any of the medically important fungi [14, 15]. Surprisingly, a positive influence of antibody against disseminated fungal disease was first suggested more than 50 years ago [16]. About 15 years later, an interesting work by Pearsall and coworkers again sensitized the scientific attention on the benefic effects of passive serum transfer for murine candidiasis [17].

In recent years several studies have established the potential efficacy of humoral immunity in host protection against *Candida albicans*, and there is a great deal of attention on the benefits that may therefore result from mAb-based therapy against various fungal infections including *Candida* infections [18]. 

Until today, in the field of clinical mycology, a single mAb able to bind a specific cryptococcal antigen in serum of patients suffering from cryptococcal meningitis has been studied clinically [23].

Candidal diseases are often chronic, difficult to treat, and carrying a high mortality and morbidity despite antifungal therapy. Invasive candidiasis is a promising area for mAb therapy because current therapies are inadequate. Usual treatment for invasive fungal infections consists in monotherapy based on the use of azoles, echinocandins, and the polyene amphotericin B (AMB) or one of its liposomal derivatives. However, the well-known toxicity of antifungal therapy and the emergence of the increasing resistance to these antifungal agents actually represent a potential problem [24, 25]. Considering this scenario, it is reasonable to assume that in the next few years, efforts to increase the antifungal therapies may also be targeted to the field of antibodies-based therapies. Several studies from the first decade of the 80s have focused on the production and characterization of monoclonal antibodies directed against candidal cell surface determinants. After the development of the hybridoma technology [26], many research groups have used *Candida* antigens for the selection of murine mAbs with diverse specificity. Findings related to such *Candida* mAbs have brought interesting observations on the variation of antigen expression by this organism [27, 28].

To date, there are some antibody molecules with more or less demonstrated efficacy in the therapy of systemic candidiasis in animal model, but all of them derived from mice [29–31] with the only exception for Mycograb. Mycograb (NeuTec Pharma, a subsidiary of Novartis AG, Basel, Switzerland) is a human recombinant antibody directed against *Candida* Hsp90 that is essential for yeast viability. This antibody has been designed to work in combination with the best current antifungal therapeutics and entered in a multinational phase III clinical trial. This paper will review Mycograb in its most salient features, with a particular focus for its variant named Mycograb C28Y.

### 1.1. Mycograb and Its Target Hsp90

The history of Mycograb antibody has distant origins. Its selection as a single chain antibody takes advantage from biotechnologies related to the expression of human antibodies as soluble recombinant proteins in *Escherichia coli* that offers the periplasmic space as an ideal site for the formation of disulfide bridges, essential for the proper folding of the antibody molecules. Mycograb derived from a cDNA coding antiheat shock protein 90 (Hsp90) antibody of patients recovered from invasive candidiasis. It consists of the variable ends of the heavy (VH) and light (VL) chains from one arm of the antibody. These two N-terminal domains are linked together by 2 cross-chain cysteine bonds with a synthetic linker to represent the antigen-binding domains. 

Resultant recombinant protein is a polyhistidine-tagged single chain antibody fragment against the immunodominant epitope of *Candida *Hsp90 antigen. Its original name, Efungumab, was converted by NeuTec Pharma in Mycograb. It is expressed in *E. coli *and easily purified by three-step chromatography, filter sterilized and lyophilized [32]. It was developed by NeuTec Pharma, in Manchester, UK, and is actually produced to current good manufacturing practice standards, by 1000 liter batch fermentation of recombinant *E*. *coli.* The clinical motivation behind the choice of Hsp90 as a target for the generation of an inhibitory antibody was based on the observation that the antibody response in patients with invasive candidiasis, receiving AMB, showed a strict correlation between recovery and antibody titer to the immunodominant Hsp90 [33]. Mycograb mimics this naturally occurring inhibition of Hsp90 and is thus a logical partner in combination therapy. Numerous studies examining the antibody response to *C. albicans* in infected patients and experimentally infected animal have demonstrated divers and specific immunodominant antigens [34].

The molecular chaperone Hsp90 is a key cellular regulator that is critical for setting cellular responses to a wide variety of stressful stimuli, among which, drug-induced stress. The essential role in cell physiological mechanisms makes Hsp90 indispensable for yeast viability.

Hsp90 regulates the stability and function of diverse client proteins [35], like its downstream effector calcineurin, a protein phosphatase that regulates the response to stress. Calcineurin dependent stress responses are required to survive to fungistatic antifungal drugs exposure, like azoles and echinocandins for *C. albicans. *Prejudice Hsp90 or calcineurin function converts antifungal drugs from fungistatic to fungicidal and enhances the efficacy of antifungals in mammalian models of systemic and biofilm fungal infections [36]. Combination therapy with antifungal drugs and Hsp90 inhibitors may therefore provide a powerful strategy to treat life-threatening fungal infections. In addition to its normal cytoplasmic localization, extracellular or membrane bound Hsp90 elicits an immune response providing a link between innate and adaptive immunities [37]. In effect, Hsp90 represents an important target for protective antibodies in disseminated candidiasis. An antibody response to Hsp90 antigen is significantly more common in patients with deep-seated candidiasis than those with superficial candidiasis [38–40]. In patients with severe, invasive candidiasis, a sustained antibody response to this antigen correlated closely with a good prognosis, whereas lack or falling levels of Ab were associated with fatality [41]. Dissecting this potentially protective antibody response to the level of individual epitopes showed that it was primarily directed against the epitope NKILKVIRKNIVKK. Mice vaccination with an Hsp90-expressing DNA vaccine demonstrated specific humoral immunity associated with protection against invasive candidiasis [42]. Deepening studies on antibody response to specific epitopes of this antigenic target led to the identification of peptides representing the epitopes LKVIRK or DEPAGE derived from the middle domain of Hsp90 able to stimulate a protective immune response against *Candida* infection [43]. Mycograb directly binds the middle domain of Hsp90 inhibiting communication between the terminal domains with client proteins [44]. The antibody is not able to cross the fungal cell wall to enter the fungal cytosol and interact with extracellular or membrane bound Hsp90. The antibody is composed of light and heavy chains without the Fc domain, abrogating the need to recruit the cellular immune system for its function and allowing efficacy even in immunocompromised patients. The antifungal activity of Mycograb can be shown using conventional assays for evaluation of antifungal drugs such as checkerboard and time kill methodologies.

The Fractional Inhibitory Concentration (FIC) is a mathematical expression of the effect of the combination of antimicrobial agents. Checkerboard titration assays result in a number of FIC indices (FICIs). The sum of a number of FICIs divided by the number of indices is designated as average ΣFIC. The synergism-antagonism FICI accepted criteria are traditionally been defined as follow: FICI value of <0.5 was defined as synergistic, a value of >0.5 and <4.0 was defined as indifferent, and a value of >4 was considered antagonistic. Other works reported as synergistic FICI values ranging from 0.5 to 1 [45].

### 1.2. Mycograb: *In Vitro* and *In Vivo* Efficacy

*In vitro* assays developed by Matthews et al. [19], for efficacy evaluation of Mycograb, showed intrinsic antifungal activity against the most important species of *Candida, *including fluconazole sensitive and fluconazole resistant strains of *C. albicans. *The Mycograb MICs were found to be rather high, ranging from 128 to 256 *μ*g/mL. Moreover, Mycograb, even at relatively high concentration (100 *μ*g/mL) demonstrated indifference when administered in combination with fluconazole against all yeasts examined. The FICI values obtained were all >0.5, with the only curious exception of the fluconazole-sensitive strain of *C. albicans *for which there was synergy with a FICI value of 0.34. By contrast, authors demonstrated *in vitro* synergistic action of Mycograb with AMB. Results from checkerboard methods showed a pronounced synergy between AMB and Mycograb against all Candida strains tested, with relatively low concentration of Mycograb of 4 or 8 *μ*g/mL readily achievable in humans. Resultant FICI values varied from 0.09 to 0.31.  Table 1 summarizes the *in vitro* interaction of Mycograb with diverse antifungal agents. After such encouraging findings derived from *in vitro* experiments, results from the *in vivo* assessment, in a normal immune mouse model of systemic candidiasis, demonstrated that a single dose of Mycograb of 2 mg/kg in combination with AMB improved the killing of each *Candida* species examined compared with AMB monotherapy [19]. AMB alone cleared the *C. tropicalis *infection but not *C. albicans, C. krusei, C. glabrata*, or *C. parapsilosis *from one or more organs. Mycograb combined with AMB resulted in complete resolution of *C. albicans, C. krusei, *and *C. glabrata,* but for *C. parapsilosis *even though the liver and spleen were cleared, the kidney colony counts were not. Furthermore, statistically significant reduction in the mean organ colony count from the same mouse model of systemic candidiasis was obtained with Mycograb alone, demonstrating an *in vivo* intrinsic antifungal activity of this antibody at a dose of 2 mg/kg. This first preclinical study demonstrated that Mycograb has synergy against a broad range of *Candida *species in combination with AMB *in vitro* and in animal models of invasive candidiasis.

### 1.3. Mycograb: The Clinical Trials

The first clinical trial of Mycograb [33] in the treatment of fungal infections in combination with antifungal agents involved five patients with invasive candidiasis in an open label tolerance and pharmacokinetic study. A test dose of Mycograb 0.1 mg/kg was given to patients before the start of liposomal AMB (L-AMB) therapy. Subsequently patients received two further doses of Mycograb at 1 mg/kg 8 or 12 hours (h) apart. The highest concentration of Mycograb 30 minutes after the first dose ranged from 1.5 to 4 mg/L, and serum levels were undetectable by 8 h. Despite too limited number of patients involved in the trial, no treatment related adverse events were noted by the investigators.

The most important medical trial in terms of clinical efficacy of Mycograb in the treatment of invasive candidiasis is a double-blind, randomized study conducted by Pachl et al. [46] to determine whether L-AMB plus Mycograb was superior to L-AmB plus placebo in 139 adult patients with invasive candidiasis. Among 139 patients, 117 were included in the modified intention to treat group. Enrollment criteria included one or more positive cultures showing candidiasis from the blood or from a deep normally sterile site but not from respiratory secretions, oropharyngeal specimens, or esophageal specimens. L-AMB was preferred by the research group instead of conventional desoxycholate form for its superior safety profile. A rigorous and sophisticated statistical analysis plan was created prior to unblinding by Hartington Statistics and Data Management (London, UK). 

The trial was conducted in 26 institutions across Europe and the United States. Patients were stratified into groups on the basis of *Candida *species *(C. albicans *versus *C.* non-*albicans) *and were randomly assigned to receive either intravenous Mycograb (1 mg/kg) or placebo (saline) every 12 h for 5 days. In addition, each patient was treated, for a minimum of 10 days, with the manufacturer's recommended dose of L-AmB. The Mycograb group included 63% *C. albicans *versus 65% in the placebo group. The two groups were besides well balanced with respect to the APACHE II scores (Acute Physiology and Chronic Health Evaluation II).

For the assessment of the efficacy of combination therapy, both mycological and clinical responses were considered. The primary efficacy endpoint was overall response to treatment on day 10, defined as a complete clinical response with resolution of all signs and symptoms of candidiasis and mycological response with negative cultures.

Secondary endpoints were clinical response at day 10, mycological response at day 10, and rate of mycological clearance of infection and *Candida*-attributable mortality four weeks (day 33) after last administration of Mycograb or placebo. Signs and symptoms of infection as well as culture results were carefully controlled by local investigators. In the meantime, trial's safety, in terms of adverse events to drugs therapy, was monitored by an independent committee. 

Side effects like back pain and vomiting, generalized rash, hypertension, and others revealed that episodes of hypertension occurred more frequently in the Mycograb group (7.4%) than in the placebo group (2.9%) and usually occurred within 2 h after receipt of the first dose of Mycograb.

Authors of the study consider, however, that Mycograb was well tolerated.

A complete overall response by day 10 was obtained for 29 (48%) of 61 patients in the L-AmB group, compared with 47 (84%) of 56 patients in the Mycograb combination therapy group. Moreover, patients who received Mycograb cleared their infections twice as quickly and importantly the Candida-attributable mortality rate decreased from 18% to 4% (>4-fold) among patients receiving Mycograb. 

Mycograb in combination with L-AMB produced significant clinical and culture confirmed improvement in outcome of patients with invasive candidiasis.

These very promising results have attracted the attention of the scientific community, and some case reports have been published on the use of antifungal agents in combination with Mycograb for treatment of severe, also pediatric, cases of disseminated *C. albicans* infections [47–49].

### 1.4. Mycograb and Caspofungin

A study of Hodgetts et al. [20] demonstrated that Mycograb increased the susceptibility of Candida to caspofungin. Echinocandins have recently become popular as an alternative to AMB in the treatment of fungal infection. Caspofungin demonstrated a more conducive safety profile than AMB, with significantly less treatment breaks due to drug toxicity [50].

In the *in vitro* experiments performed in this study, caspofungin and Mycograb concentrations were tested alone and in combination by MIC endpoints and checkerboard titrations (Table 1). Results from susceptibility testing demonstrated that the addition of Mycograb improved the susceptibility to caspofungin of a variety of isolates that represent the most important species causing invasive candidiasis. Wisely, Hodgetts and collaborators tested both endpoints and assessed the combination of caspofungin and Mycograb in the same mouse model used by Matthews et al. in the preclinical assessment of the efficacy of Mycograb to outline its synergy with AMB. Efficacy in mice was measured 48 h after the intravenous injection of the yeasts by the reduction in mean colony counts from kidney, liver, and spleen or the number of negative biopsies as appropriate. The enhanced activity of combination therapy (4 mg/kg caspofungin plus 2 mg/kg Mycograb) compared with monotherapy with 4 mg/kg caspofungin achieved statistical significance against a variety of Candida isolates. Such convincing data support the hypothesis that the addition of Mycograb to caspofungin could improve outcome in a way similar to that seen with L-AMB therapy. Authors of the study rightly point out the involvement of Hsp90 in the mechanisms of Candida resistance to caspofungin [51], assuming a contribution, in the overall treatment success of this combination therapy, and in increasing yeasts susceptibility by Mycograb.

### 1.5. Mycograb and CHMP

In November 2006, the Committee for Medicinal Products for Human Use (CHMP) adopted a negative opinion, recommending the refusal of the marketing authorization for the medicinal product Mycograb. The motivations were related to quality aspects and safety concerns.

The quality concerns included incoherence in the structure of the compound between manufactured batches, such as the way the molecules of Mycograb may fold or aggregate in the solution for injection and the level of some substances that could stimulate an immune response in patients. Safety concerns were associated with “cytokine release syndrome” a condition that can cause nausea, vomiting, pain, and also hypertension. 

In March 2007, following the reexamination, the CHMP removed their concern regarding the cytokine release syndrome and hypertension, as these would be manageable in clinical practice. However, all other concerns due to heterogeneity, including autoaggregation of the mAb, remained, hence, the CHMP confirmed the refusal of the marketing authorization on 20 March 2007.

### 1.6. Mycograb C28Y Variant

In response to the failure of marketing authorization by the CHMP, Arnold Louie and collaborators [21] attempted to overcome the issues related to the heterogeneities in molecular weight and conformational structure of Mycograb. Authors believed that autoaggregation of the molecule could be due to the presence of a fifth cysteine at position 28 which was unpaired. This inconvenient amino acid was not in the antigen-binding site of the antibody fragment; therefore it was not implicated in the interaction with target Hsp90 and did not contribute to the two disulfide bridges normally present in the molecule. Consequently, a modified form of Mycograb named as Mycograb C28Y variant was developed in which the cysteine (C) at position 28 was changed to a tyrosine (Y).

A polar uncharged r group, with similar physical and chemical properties, characterizes both amino acids. Such amino acid substitution should not modify the antibody conformational structure, and therefore, new amino acid composition should not interfere with target interaction. Data sets from Novartis on the quality of new formulation has indicated that the Mycograb C28Y variant appeared to be much more stable and homogeneous, with a markedly improved batch-to-batch consistency and an ability to be dissolved in solution as monomeric form equal to 80% of the preparation.

First data on MIC for the Mycograb C28Y variant, as a single agent, was >256 *μ*g/mL. Higher concentrations could not be tested because concentrations of ≥512 *μ*g/mL became turbid. 

This turbidity could be due to partial recombinant antibody precipitation as a result of physical and chemical conditions not favorable for those concentrations that have consequently induced aggregation. However, concentrations of 0.5 mg/mL are not particularly high to justify this phenomenon that still regards the largest part of recombinant proteins expressed in *E. coli*.

Moreover, in contrast to the synergistic *in vitro* and *in vivo* interactions that have been demonstrated between Mycograb and AMB by Matthews et al., these new sets of experiments performed by multidose treatment studies in a neutropenic murine model of systemic candidiasis showed indifference between the Mycograb C28Y variant and AMB. The efficacy of Mycograb C28Y variant combined with AMB was not better than AMB monotherapy in clearing *C. albicans* from the kidneys, livers, and spleens of infected mice. Again, in neutropenic mice Mycograb C28Y variant alone had no intrinsic activity against *C. albicans*. 

Surprisingly, FICI values describing the synergistic *in vitro *interaction between Mycograb C28Y variant and AMB were similar and consistent with prior data for Mycograb and AMB. Comparison of FICI values for both mAbs is reported in Table 2. The discordance between the synergistic interaction between the Mycograb C28Y variant and AMB observed in the checkerboard assay and lack of the outcomes observed in neutropenic infected mice was not expected. A very detailed discussion of what may be the motivations that could explain the different *in vivo* interactions observed for Mycograb, and the Mycograb C28Y variant is provided in the work of Arnold et al. First point under consideration concerns the compounds themselves as there may be a difference in potency of the Mycograb C28Y variant compared to Mycograb. However, the modification of the structure of a compound is frequently associated with pharmacokinetics changes of the molecule, and pharmacokinetic studies conducted by the group in neutropenic mice have definitively ruled out this hypothesis. With regard to the animal models, original Mycograb formulation was evaluated in normal immune mice and in predominately nonneutropenic human patients, while Mycograb C28Y variant was used alone and in combination with AMB in a neutropenic murine model of systemic candidiasis. Farther, authors opted for a multidose treatment design study, instead of single doses of AMB and Mycograb administered by Matthews et al. to evaluate the potential therapeutic benefit of the Mycograb C28Y variant in order to build upon a phase III human trial which normally used a multidose treatment regimen.

Regarding different animal models, authors reported data by others (data from Novartis) on the efficacy of the Mycograb C28Y variant and AMB in immunocompetent mice. The new Ab formulation provided no benefit over AMB monotherapy. Afterwards the authors discuss the possibility of an interference due to the production of anti-human antibody by mice with normal immune systems in response to receiving multiple injections of this human recombinant Ab fragment. The concentration of the Mycograb C28Y variant in mice serum was not measured at the time the multidose study was conducted as a validated test was not available.

The single-dose and multidose pharmacokinetics of the Mycograb C28Y variant in the neutropenic murine model of systemic candidiasis used by Arnold et al. were similar to those obtained for the original Mycograb formulation, suggesting that the neutropenic mice did not produce anti-human antibody against the Mycograb C28Y variant in response to multiple administrations of this compound. Finally, differences in the mouse strains used to evaluate the activities of the original Mycograb formulation and the Mycograb C28Y variant could not explain the difference in activity of the two molecules. It is clear from this investigation that important considerations should be carefully evaluated on the limits of *in vitro* checkerboard assay for the effective estimation of *in vivo* efficacy and synergism with other drugs, of a testing molecule. Retrospectively, previous works missed some important test controls, such as parallel evaluation of *in vitro* antifungal properties of unrelated similar recombinant antibodies. 

 Work from Richie and collaborators [22] did not miss such relevant *in vitro* controls and at least clarified ambiguous results arising from the checkerboard assay. 

In this study, done in a checkerboard design, combinations of AMB and Mycograb C28Y (up to 128 *μ*g/mL) caused a dose-dependent decrease of 2 to 3 dilution steps in the MIC of AMB against two strains of *C. albicans*, in agreement with previous reports.

Unrelated proteins, including 2 murine IgGs, human gamma globulin, bovine serum albumin, and human serum, were tested in parallel as protein controls. All unrelated proteins, with the exception of human serum, at 5 *μ*g/mL, produced a 4-step dilution reduction in the MIC of AMB against *C. albicans*. Human serum showed a paradoxical effect with a 3- to 5-step dilution reduction at concentrations up to 5% and a 1-step dilution reduction at concentrations above 10%.

Results from this study demonstrated that small amounts of serum present in RPMI medium can potentiate the activity of AMB which is attenuated at higher concentrations of serum.

To independently validate their findings, similar experiments were performed by other investigators in a Medical Mycology department belonging to a different University. Microdilution assays were performed in checkerboard methods against two strains of *C. albicans* to evaluate the antimicrobial activity of AMB in combination with various protein sources. This included human gamma globulin and serum albumin, the original Mycograb, Mycograb C28Y, and Aurograb, a similar recombinant antibody fragment designed to bind to an unrelated bacterial target [52]. All proteins tested in combination with AMB recorded a FICI value <0.5, indicating a synergistic relationship (Table 2). Interestingly, the nonspecific, synergistic protein effect was not observed in combination studies with fluconazole or caspofungin, attributing to Hodgetts work an indirect greater validity.

## 2. Conclusion

The growing increase of drug resistance in fungal pathogens compromises the efficacy of the limited number of antifungal drugs available to date. Furthermore, antifungal drugs possess a limit number of distinct targets. Fungi are eukaryotes, and the close evolutionary relationships of these opportunistic pathogens with their human hosts make most treatments toxic to the host or weak in combating infections. The use of drug combinations has emerged as a powerful strategy to enhance antifungal efficacy and abolish drug resistance; even though the impact on the evolution of antifungal resistance remains largely unexplored and unresolved. Combination therapy has the potential to play down the evolution of drug resistance by more effectively eradicating pathogen populations and by requiring multiple mutations to confer drug resistance. *In vitro *and first clinical data showed that Mycograb owns activity against *Candida *spp. when used alone and synergism when combined with AMB, fluconazole, and caspofungin. Important studies reviewed in this paper suggest that the checkerboard assay does not predict the *in vivo* interaction between Mycograb, its variant C28Y, and AMB. The antifungal potentiation of AMB by Mycograb *in vitro *appears to be a nonspecific effect that can be reproduced by a wide range of unrelated proteins. Therefore, the *in vitro* checkerboard assay cannot replace *in vivo* studies in assessing the interaction of anti-Hsp90 antibody formulations with AMB for the treatment of invasive Candida infections. In this respect, although further confirming data are needed, it looks like that Mycograb may become a new antifungal agent with unique mechanism of action for treatment of invasive candidiasis.  Table 3 shows in a very simplified manner the results of the *in vitro* and *in vivo* efficacy of Mycograb and its variant C28Y in combination with AMB. Results from clinical trials on adult patients with invasive candidiasis remain, anyway, very encouraging. Currently, Europe's Committee for Medicinal Products for Human Use (CHMP) refused approval of Mycograb due to a lack of data concerning adverse effects, specifically the cytokine release syndrome characterized by hypertension, nausea, and vomiting which were handled by corticosteroids and antihistamines. CHMP requested more data from a controlled trial to clarify the nature of the cytokine release syndrome. Mycograb has orphan drug status with the Food and Drug Administration (FDA) and is available on a compassionate use basis in Europe. Future directions for Mycograb include adjunctive therapy of cryptococcal meningitis where it has shown synergistic activity with AMB and fluconazole [53]. Identification of protective mAbs against fungi may be useful for both the development of direct Ab-based therapies and isolation and characterization of defined antigens able to elicit protective Ab immunity. The challenge in constructing antibody-based antifungal vaccines is to identify the fungal antigens which elicit protective antibodies response and to develop strategies to direct the antibody response towards the production of effective natural antibodies while avoiding the production of nonprotective or deleterious antibodies [54]. It would be of great impact for medical mycology to take advantage of the available technologies for the selection of human mAbs to strengthen the treatment of invasive fungal infections by combination therapies. Phage display antibody library methodology represents an excellent tool for dissecting the humoral immune response of patients with invasive candidiasis. The enormous advances in the field of proteomics now allow the identification of relevant immunodominant targets closely related to the clinical course of infectious disease [55]. Due to all its multiple antibody specificities, no other biological sample can give as much information as patient's serum about the interaction and fight between host and pathogen. Huge potentialities associated with the use of antibody library on the surface of filamentous phages [56–59] could in part allow to explore such different antibodies specificity by selecting single human monoclonal antibodies against Candida protective antigens.

## Figures and Tables

**Table 1:** Checkerboard assay of FLC (fluconazole), AMB, caspofungin, and Mycograb versus *C. albicans* fluconazole-susceptible (FLC-S) and fluconazole-resistant (FLC-R) strains.

Straints	Agents	MIC (*µ*g/mL) for each agent		FICI	Outcome	References
		Alone	In combination			
*C. albicans* (FLC-S)	FLC Mycograb	1.56 128	0.4	0.34	Synergy	Matthews et al. 2003 [19]
*C. albicans* (FLC-R)	FLC Mycograb	50 256	12.5 100	0.64	Indifference	
*C. albicans* (FLC-S)	AMB Mycograb	1 128	0.03 8	0.09	Synergy	Matthews et al. 2003 [19]
*C. albicans* (FLC-R)	AMB Mycograb	0.5 256	0.125 4	0.27	Synergy	
*C. albicans* (FLC-S)	Caspofungin Mycograb	0.125 1024	0.0625 16	0.5	Synergy	Hodgetts et al. 2008 [20]
*C. albicans* (FLC-R)	Caspofungin Mycograb	0.25 2048	0.125 0.125	0.52	Indifference	

FICI: fractional inhibitory concentration index.

**Table 2:** FICI of AMB in combination with Mycograb, Mycograb C28Y variant, and various protein sources.

*C. albicans* STRAINS	AGENT	FICI	Outcome	References
ATCC 24433	AMB MYC C28Y	0.258	Synergy	Louie et al. 2011 [21]
ATCC 90028	AMB MYC C28Y	0.258	Synergy	Louie et al. 2011 [21]
ATCC 24433 ATCC 90028	AMB MYC C28Y	0.27 ± 0.18	Synergy	Richie et al. 2012 [22]
ATCC 24433 ATCC 90028	AMB Mycograb	0.23 ± 0.12	Synergy	Richie et al. 2012 [22]
ATCC 24433 ATCC 90028	AMB Aurograb	0.14 ± 0.01	Synergy	Richie et al. 2012 [22]
ATCC 24433 ATCC 90028	AMB Human *γ*-globulin	0.18 ± 0.07	Synergy	Richie et al. 2012 [22]
ATCC 24433 ATCC 90028	AMB Human-serum albumin	0.15 ± 0.05	Synergy	Richie et al. 2012 [22]

FICI: fractional inhibitory concentration index.

**Table 3:** *In vitro* and *in vivo* final evaluations of the efficacy of Mycograb and its C28Y variant.

	*In vitro *		*In vivo *	
	Intrinsic fungicidal activity (MIC)	Combination therapy with AMB (CB)	Intrinsic fungicidal activity (MIC)	Combination therapy with AMB (I.C.)
Original Mycograb	present	synergy	present	effective
Mycograb C28Y Variant	absent	synergy	absent	non effective

(CB): checkerboard assay; (I.C.): invasive candidiasis on a mouse model of systemic infection; (MIC): minimal inhibitory concentration.
